# Clozapine-Induced Pericardial Effusion Presenting with Persistent Tachycardia

**DOI:** 10.1155/2021/5523562

**Published:** 2021-08-28

**Authors:** Nathan Gilbreth, Hari Nath, Fernando Quesada, Delatre Lolo

**Affiliations:** ^1^MD Candidate, Class of 2022, New York Medical College, 40 Sunshine Cottage Rd, Valhalla, NY 10595, USA; ^2^Internal Medicine Resident, New York Medical College-Metropolitan Hospital Center, 1901 1st Avenue, New York, NY 10029, USA; ^3^Internal Medicine-Metropolitan Hospital Center, 1901 1st Avenue, New York, NY, 10029, USA

## Abstract

Clozapine is an atypical antipsychotic used in refractory schizophrenia and depression. Its use is often complicated by its vast side-effect profile including cardiovascular reactions, agranulocytosis, and seizures. Specifically, the cardiac complications of clozapine have been shown to predominantly cause myocarditis and pericarditis. In this case report, the case of a 58-year-old male being treated for treatment-resistant depression and schizophrenia who suffers from tachycardia is presented. He is treated empirically for orthostatic hypotension with IV fluids without much success. Further imaging and echocardiography demonstrated a pericardial effusion, a rare reaction (≤1 : 10000) that has only been documented in a handful of case reports. This anecdotal evidence highlights the significance of polyserositis/pericardial effusion in the context of clozapine-induced orthostatic hypotension resistant to rehydration. When starting a patient on clozapine, it is important to consider further workup and monitoring with laboratory baseline biomarkers and cardiac evaluation with symptomatic individuals. Upon immediate cessation of clozapine, the pericardial effusion should spontaneously resolve without complication and should not be rechallenged.

## 1. Introduction

Clozapine has been significant in its efficacy as an antipsychotic drug for treatment-resistant psychotic depression [1]. Despite its lower risk of tardive dyskinesia and extrapyramidal symptoms, its use as an effective agent has been limited and complicated by its adverse side-effect profile. The most severe side effects include agranulocytosis, respiratory depression, and seizures [2]. However, there have been multiple uncommon adverse cardiovascular effects noted with its use, including hypotension, cardiac conduction abnormalities, tachycardia, myocarditis, polyserositis, and pericarditis [3, 4]. In particular, there have been very few reports of clozapine-induced pericardial effusion; hence, it is often disregarded as a potential etiology. In this report, we present a 58-year-old male with a history of schizophrenia who is treated with clozapine and has a subsequent moderate pericardial effusion and orthostatic hypotension. We will discuss the initial clinical management and diagnostic rule out during admission, as well as the resolution with immediate discontinuation of the medication.

## 2. Case Presentation

A 58-year-old Caucasian male with a past medical history of hypertension, schizophrenia, alcohol abuse, and panic disorder was admitted to inpatient psychiatry with symptoms of suicidal ideation and auditory hallucinations. He had failed trials of multiple antidepressants and antipsychotics. A month into his admission, due to treatment-resistant depression, he was started on clozapine 100 mg PO BID and lithium 300 mg PO daily. 12 days after the initiation of the medications, he developed the new onset of tachycardia ranging between 110–130s bpm and had a brief febrile episode of 102.5 F a couple days later. He underwent a U/A which showed cloudy urine with moderate leukocyte esterase, white blood cells 15–30, and few bacteria. The patient at the time did not note any dysuria or other urinary complaints.

**Figure 1:**
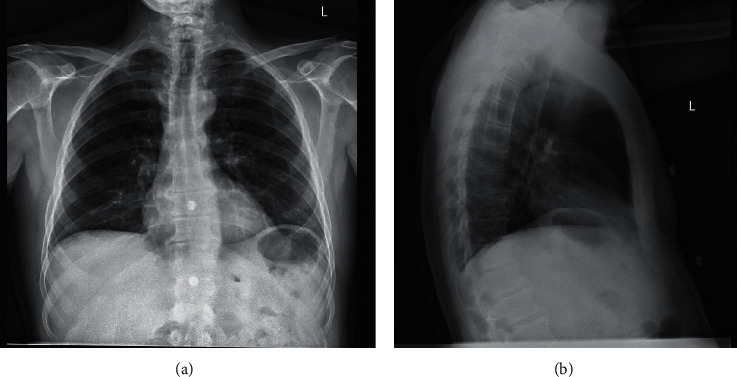
Frontal (a) and lateral (b) chest X-ray images demonstrating no abnormalities or signs of pericardial effusion.

**Figure 2:**
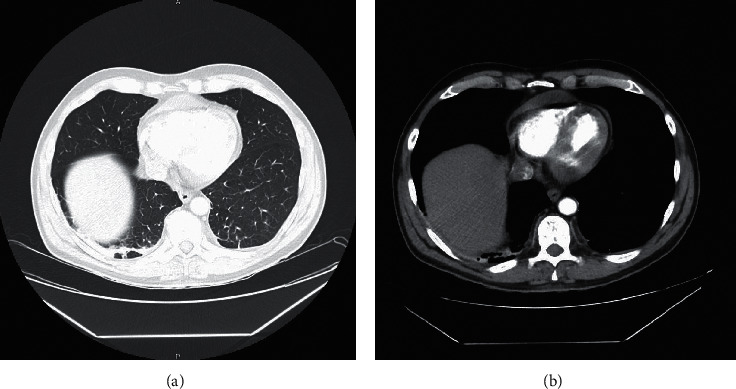
CTA chest with (a) and without (b) contrast enhancement demonstrating the moderate pericardial effusion. No pleural effusion or other abnormalities are present.

He was initially recommended admission and started oral ciprofloxacin, which was immediately switched to IV ceftriaxone x3 days for a presumptive diagnosis of UTI. At initial evaluation, he was empirically treated with a bolus of IV fluids for orthostatic hypotension (supine 115/72, upright 103/59 mmHg). On admission, chest X-ray demonstrated no acute findings (Figure 1) and EKG demonstrated sinus tachycardia at a rate of 109 bpm with low-voltage QRS. His basic metabolic panel, TSH, fT4, and complete blood count were unremarkable as well. During his stay, he continued to have persistent tachycardia with a peak of 153 bpm despite completing the antibiotic regimen and IV fluids. Given his persistent tachycardia and concern for thrombus, he underwent a CTA chest (Figure 2) with contrast which demonstrated moderate pericardial effusion with subcentimeter bilateral hilar lymph nodes. Given the low-voltage QRS findings, he also had a TEE performed which demonstrated grossly normal LV function with small pericardial effusion present, without evidence of tamponade. A repeat EKG demonstrated similar sinus tachycardia at a rate of 118 bpm with low-voltage QRS. For the duration of admission, he had no complaints of chest pain, shortness of breath, or discomfort.

There was a discussion with the psychiatry team regarding concerns for the vast side effects from recently prescribed clozapine vs. moderate dehydration. He received more IV fluids, and clozapine was promptly discontinued. Infectious disease was consulted, and further workup demonstrated negative coxsackie B but a positive coxsackie A virus with titers 1 : 800. This was a confounding etiology for the pericardial effusion, despite limited literature supporting coxsackie A as a cause for a pericardial effusion. Blood and urine cultures were negative at the time as well. Although after discussion, alternative etiologies such as SLE, uremia, or malignancy were considered, limited clinical suspicion rendered further workup unnecessary at that time. Given the subacute presentation in the setting of a probable offending medication, idiopathic etiology was deemed less likely.

Only 3 days after discontinuation of clozapine, his tachycardia had spontaneously resolved, and a repeat EKG showed a resolved normal sinus rhythm at 91 bpm with normal QRS voltage. No treatment of the pericarditis was initiated given the presumed cause related to a medication adverse effect. His vitals continued to remain stable, and he was subsequently discharged home.

## 3. Discussion

Clozapine is an atypical antipsychotic which is often used as a last resort in treatment-resistant schizophrenia. The dangerous side-effect profile includes agranulocytosis, seizures, respiratory depression, and a multitude of cardiovascular complications [2, 4]. This particular case is of interest, as there are only a handful of reported cases of pericardial effusion (polyserositis) and fewer still with concomitant orthostatic hypotension. According to the American College of Cardiology, there is *a* ≤ 1 : 100 risk of suffering from the adverse effect of orthostatic hypotension. Despite myocarditis/pericarditis being ≤1 : 10000 risk, pericardial effusion was still not established as an adverse outcome [4]. As per French imputability of casualty assessment, this reaction received a designation of C1S3I2B2. This case report highlights the importance of careful cardiac monitoring in patients that are taking clozapine.

There are no published guidelines for managing and monitoring associated cardiomyopathy, myocarditis, and pericarditis outcomes from clozapine. However, there is well-documented evidence that markers including CRP, ESR, troponins, BNP, and echocardiography are all potentially useful in evaluating a baseline prior to regimen initiation [4]. In our case specifically, inflammatory markers including ESR and CRP were not obtained. Having baseline reference labs could have expedited the prediction of the underlying etiology of pericardial effusion/pericarditis. In addition, leukocytosis is another marker of pericardial syndrome. This was not identified during our patient's hospital course, despite evidence of multiple other case reports with these findings [4, 5]. Some studies have shown the onset of eosinophilia may also predict cardiac outcomes associated with clozapine [4]. In patient outcomes of sudden cardiac death, there were found to be cardiac eosinophilic infiltrates with myocytolysis, attributed to a hypersensitivity drug reaction [6]. However, in this report, eosinophils in serum remained stable and within normal limits for the duration of admission. Despite strong evidence for using laboratory biomarkers in identifying a pericardial complication, they may not always be present. This may necessitate the use of further imaging studies to confirm a diagnosis.

During presentation of subacute tachycardia without any apparent pathology, in the context of clozapine initiation, a more robust cardiac evaluation with necessary imaging is likely to be beneficial. Given that the adverse effect of pericardial effusion can manifest at any point, from one week to years after treatment initiation, serious considerations for monitoring should be taken [7]. By obtaining a TEE early in the treatment course, we were able to obtain a timely and definitive diagnosis. This allowed for prompt discontinuation of clozapine and helped avoid any severe forms of pericardial tamponade, a potentially fatal complication. Spontaneous resolution of the effusion, as seen in our case, can also be seen in many other studies [4, 7, 8]. The 9.1 to 17.4 hr half-life of clozapine corresponds well with the several-day resolution seen following cessation of the drug [9].

The mechanism of the various clozapine-associated cardiovascular side effects is not well understood. Researchers have suggested sympathetic hyperactivity, decreased vagal tone, and cholinergic and adrenergic inhibition as potential causes. Since there is a lack of trials to demonstrate these pathophysiologies, studies that further elucidate the adverse effects and potential intervention and prevention strategies will be particularly valuable [10].

Following cessation, recommendations include careful supportive care only [11]. Follow-up EKG has been noted in prior studies, but despite lack of an evidence-based criterion, this is at the provider's discretion [7]. A literature review found that patients rechallenged with clozapine after agranulocytosis and myocarditis had poorer outcomes [12].

An important takeaway from this case was that nonspecific tachycardia in the context of recent clozapine initiation should be closely monitored for an extended spectrum of cardiac toxicity. This should include preliminary laboratory markers for inflammation and leukocytosis (specifically eosinophilia). If IV fluids do not resuscitate the orthostasis, prompt evaluation with TEE should be considered to allow for quick discontinuation of the offending agent, clozapine.

## Data Availability

Data are available on request.

## Abbreviations

PO: Per os (by mouth)
BID: Bis in die (twice daily)
U/A: Urinary analysis
UTI: Urinary tract infection
EKG: Electrocardiogram
IV: Intravenous
CTA: Computerized tomography angiography
TSH: Thyroid-stimulating hormone
fT4: Free thyroxine
LV: Left ventricle
BP: Blood pressure
TEE: Transesophageal echocardiogram
CRP: C-reactive protein
ESR: Erythrocyte sedimentation rate
BNP: Basic natriuretic peptide
SLE: Systemic lupus erythematosus.
